# Longitudinal changes in youth baseball batting based on body rotation and separation

**DOI:** 10.1186/s13102-023-00774-5

**Published:** 2023-11-28

**Authors:** Toshiharu Tsutsui, Jun Sakata, Wataru Sakamaki, Toshihiro Maemichi, Suguru Torii

**Affiliations:** 1 Faculty of Sport Sciences, Waseda University, Tokorozawa, Japan; 2 Toyota Athlete Support Center, Toyota Memorial Hospital, Toyota, Japan; 3 Graduate School of Sport Sciences, Waseda University, Tokorozawa, Japan

**Keywords:** Hitting, Youth athlete, Athletic performance, Biomechanics, Longitudinal study

## Abstract

**Background:**

Identifying the characteristics of batting mechanics, such as the proper angle and position of each body segment in youth baseball players, is important for proper instructions. This study aimed to identify the age-related changes in batting kinematics including rotational and separational movements of the head, upper trunk, pelvis, and arms, in youth baseball players.

**Methods:**

Over the three seasons, we measured the batting motion of baseball players aged 6- to 12 years using three high-speed cameras. Participants were divided into six age categories according to the little league eligibility rules (players were classified according to their age as of July 31 of a given year). Toss batting was performed using an automatic tossing machine set obliquely in front of the batter. Additionally, we analyzed the rotation angles of the head, upper trunk, pelvis, and arm direction, and the separation angles—calculated using the difference of each rotational angle and the head movement distance and step width—at five points in batting phase: stance, load, foot contact, pre-swing, and ball contact. Finally, 17 players from under 8 (U8, i.e., approximately 7–8 years) to U10 and 13 from U11 to U13 were analyzed. A one-way repeated measures analysis of variance was performed to analyze age-related changes in batting kinematics.

**Results:**

Several age-related changes in batting kinematics at various batting point were observed. The head-to-upper trunk separation angle increased with age from U8 to U10 during the foot contact (effect sizes [ES] = 0.658) and from U11 to U13 during the pre-swing (ES = 0.630). Additionally, the U13 showed a significantly increase in the upper and pelvis separation angles during load, foot contact, and pre–swing compared with U11 and U12 (ES = 0.131, 0.793, and 0.480).

**Conclusion:**

Various changes in batting kinematics occurred among each age group. Notably, U12 and U13 had the greater upper trunk-to-pelvis separation angle at foot contact and pre-swing compared to U11. Therefore, it would be important for the instruction of younger baseball players to understand the underdevelopment of trunk separation when batting and encourage the acquisition of such separation movements.

## Introduction

Baseball batting is one of the most complex mechanical motions in sports [1, 2], and the kinetic chain—the mechanical energy transfer from the lower limb to the upper limb through successive body segments [3] —is crucial for proper bat swing [4]. Several studies have explored the differences in batting kinematics and kinetics among different age groups or league levels to understand the development of optimum batting mechanics [5–7]. Dowling and Fleisig [6] reported that young baseball players have greater pelvic rotation angle and a higher angular velocity toward the pitcher compared to the adult professional baseball players during batting. Notably, it is suggested that the pelvic movement contributes most to the energy production required for trunk rotation in these baseball players [8]. For experienced baseball players, the batter efficiently swings the bat using the kinetic chain by transferring their weight to the pivot leg and rotating the trunk slightly to the catcher’s side during the translational phase from stance to step leg landing. Additionally, this translational phase contributes to energy accumulation for the swing and the quick rotation of the lower extremity-trunk during the subsequent rotational phase. Moreover, the growth process can impede batting mechanics because youth baseball players still growing physically have underdeveloped muscle strength, coordination, and motor function. Therefore, understanding the characteristics of batting mechanics such as the proper angle and position of each body segment in youth baseball players is vital for efficient swinging of the bat.

Nevertheless, it is difficult for younger children to separate rotational movements in each body segment. Assaiante and Amblard [9] reported that little head-to-upper trunk separation occurs during rotational movements until approximately 6 or 7 years. However, young pitchers show poor pelvic and trunk rotation timing, resulting in these segments facing the target too early during the throwing motion [10, 11]. Batting requires the simultaneous motion of both arms; therefore, it is easily affected by the turning motion. Furthermore, although it is necessary to direct the arms and the trunk toward the catcher during the translation phase, the head must remain facing the pitcher. Therefore, although difficult, acquiring separate rotational motion in each segment is vital for improving the batting performance of young baseball players. Previous cross-sectional studies [6, 7, 12] have compared the biomechanics of baseball batters with various skill levels and revealed that batting kinetics and kinematics vary according to age and skill level. Although these studies have shown differences in biomechanics among age groups, they did not identify when and how these changes occur within individuals during youth.

Hence, the present study examined the kinematic age-related changes in batting during youth by considering rotational and separational movement of the head, upper trunk, pelvis, and arms. It was hypothesized that significant individual changes in batting kinematics as the players grew older would be observed. Although each segment—head, upper trunk, pelvis, and arms—was expected to rotate in the same direction toward the pitcher or catcher side as the batting motion at younger ages, it was also expected that these players would be able to acquire separate motions with age.

## Methods

### Participants

We initially recruited 230 junior baseball players from six teams in Tokyo, Japan in April 2018. The inclusion criteria were males aged between 6 and 12 years. The exclusion criteria were injury and illness that prevented the measurements of the participant. Participants were categorized by age groups during the baseball season according to the little league eligibility rules—players were classified according to age as of July 31 of a given year. Next, the period up to July 31 of the second grade of elementary school was defined as Under 8 (U8), and after that, the period was divided by year up to U13. They played and practiced baseball for 3–6 h at least twice a week (Saturdays and Sundays). Before the examination, all participants completed a data questionnaire requesting the following information: birth, age when they first started playing baseball, and the side on which they bat. Additionally, all participants and their guardians received a detailed explanation of the experimental procedures and risks of the research before measurements were performed. Moreover, written informed consent was obtained from all participants and their guardians who assented to the study. This study was approved by the Ethics Committee of the Waseda University (No. 2018 − 208).

### Batting procedure

**Fig. 1:**
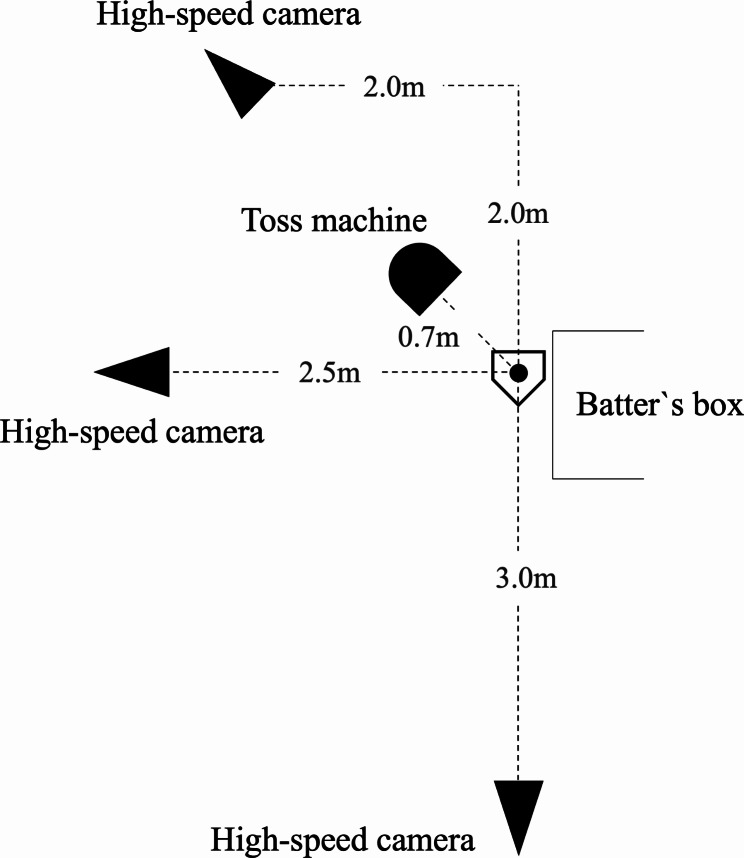
Environment of batting trial (In the case of left-handed batter)

Testing was conducted between 09:00 and 16:00 at an outdoor baseball field maintained under standard environmental conditions. In addition, testing was conducted between January and March, divided into 4 to 6 days per season. First, we measured the participant`s height and weight with their clothes on. Next, without their shoes on, height was measured to the nearest 0.1 cm without their shoes on using a stadiometer (YG200DN, Yagami Co., Nagoya, Japan); and weight was measured to the nearest 0.1 kg using a digital scale (BC622, TANITA Co., Tokyo, Japan). Next, the batting trial was performed after simple warm-up exercises, including dynamic stretching, jogging, light throwing, and swinging for approximately 20 min. Each participant received non-reflected white markers on the top of the head, both lateral acromion tips, and anterior and superior iliac spines. The trial involved toss batting with an automatic toss machine (FTM-240; Field Force Company, China). The toss machine was placed 0.7 m from the center of the home plate on the opposite side of the batter and 1.1 m toward the pitcher; it was positioned to launch obliquely in front of the batter. The height of the tossing machine was adjusted according to the participant’s height as follows: 45% of the height minus 52.5 cm. Then, actual testing was performed twice after one practice attempt, and the batting motion was captured at 240 Hz with three high-speed cameras (Ex-100PRO, Casio Co., Tokyo Japan) placed on the batter’s side, back, and front (obliquely). The environment of the batting trial setting was shown in Fig. 1.

Additionally, the swing velocity—a component of the batting performance was measured using a Zepp sensor (ZEP-BT-000002; Zepp Company, Cupertino, California, USA), which has been shown to have high reliability (ICC, 0.88) [13], and indicated to correlate moderately to strongly with data analyzed by 3D motion capture [14]. Participants were allowed to reattempt the batting trial if they missed the ball while swinging or made timing mistakes. During the batting trial, participants used the bat they would normally use in baseball practice and games and consistently used the same bat throughout their trials. Data were collected from the test with the highest swing velocity.

### Variables

The rotation angles of the head, upper trunk, pelvis, and arm direction in the horizontal plane during the batting motion and the separation angle between each segment, the amount of head movement, and the step width were analyzed by manual digitizing using a motion analysis system (Frame-Dias V; DKH, Tokyo, Japan). Moreover, we visualized the body markers attached to the head, both lateral acromion tips, anterior and superior iliac spines, nose, toes, and the midpoint between both hands on the bat on the screen using a digital format. Next, three-dimensional coordinates were obtained using the direct linear transformation method [15], and the right-hand orthogonal reference frame was defined as the X-axis, Y-axis, and Z-axis. The Y-axis was directed from the pitcher’s mound to the home plate, and the Z-axis indicated a vertical direction (bottom to top). Additionally, the X-axis was defined as the cross-product of the Y-axis and Z-axis. For calibration, poles with nine markers (from 0 to 2.0 m at 25 cm intervals) were vertically set in a 4 × 4 grid at 40 cm intervals (the standard errors were as follows: x = 0.22 cm; y = 0.28 cm; z = 0.34 cm). A recording of the calibration points using the three high-speed cameras was conducted from the start to the end of batting. The analysis data were collected at five points: stance, load, foot contact, pre-swing, and ball contact. Stance and foot contact were defined as the point of the toe of the stepping leg on the Z-axis at which the value of the Z-axis began to increase in the positive direction. Moreover, load and pre-swing were defined as the midpoints between stance and foot contact and between foot contact and ball contact, respectively.

**Fig. 2:**
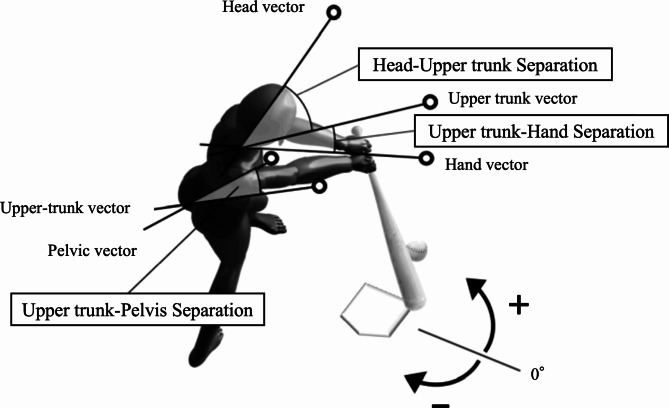
Definitions of rotation and separation variables

All rotation angles were calculated using values corresponding to spaces in global coordinates because batting is an operation initiated by reacting to a thrown ball and defined as the projected angle on the horizontal plane regarding the X-axis (Fig. 2). Additionally, the rotation angles were set as positive/negative toward the pitcher/catcher.

The variables analyzed in this study and their definitions are as follows:

- Head rotation—the angle between the head vector (top of the head to the nose) and the X-axis.
- Upper trunk rotation—the angles between the upper trunk vector (passing through the midpoint of both acromions and perpendicularly to the line connecting both points) and X-axis.
- Arm direction—the angle between the hand vector (midpoint of both acromions to a point between both hands) and the X-axis.

Furthermore, the separation angle was expressed as the difference between each rotation angle, and head-to-upper trunk separation was calculated by subtracting the head rotation from the upper trunk rotation. Moreover, upper trunk-to-arm separation was calculated by subtracting the upper trunk rotation from the arm direction. Upper-to-pelvis separation was calculated by subtracting the upper trunk rotation from the pelvis rotation. The linear head movement distance (head movement) from stance to foot contact and foot contact to ball contact was calculated as the resulting displacement of the top of the head. Lastly, the stance widths during stance and foot contact were calculated as the distance between the toes.

### Statistical analysis

**Fig. 3:**
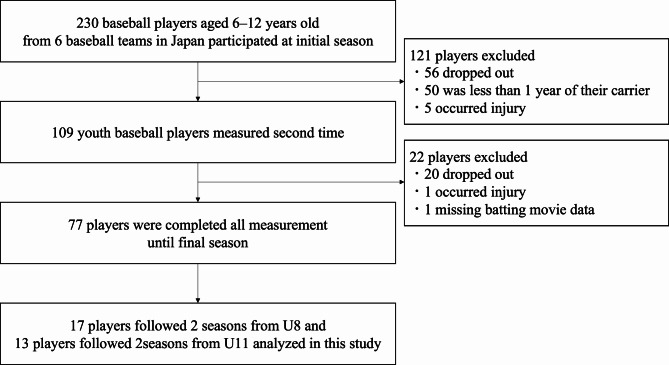
Flow diagram of exclusion criteria and the final participants

A statistical power analysis was conducted for sample size estimation. We required more than 12 players for this study to conduct a comparison of the three groups at 80% power, an alpha of 0.05. and a partial η of 0.14. Seventy-seven baseball players who met the inclusion criteria completed three measurements for three seasons. Of these, 17 players formed group 1 (U8 to U10), and 13 formed group 2 (U11 to U13) (Fig. 3).

Descriptive statistics (mean ± standard deviation) were performed. After confirming all data were normally distributed using the Kolmogorov–Smirnov test and confirming homoscedasticity with Levene’s test, we performed a one-way repeated measures analysis of variance (ANOVA) to compare chronological age, height, body weight, competition years, rotational and separation angles, swing velocity, head movement, and step width at the points of stance, load, foot contact, pre-swing, and ball contact among the initial, second, and final measurements for three seasons. Furthermore, we performed multiple comparisons of the means of the monitored variables using the Bonferroni test. Partial η^2^ was calculated for the effect size of the one-way ANOVA, with values of ≥ 0.01 to < 0.06, ≥ 0.06 to < 0.14, and ≥ 0.14, indicating small, medium, and large effects, respectively [16]. Lastly, the alpha level was set at 0.05 and all statistical analyses were performed using SPSS Statistics 27.0 (IBM, Armonk, New York, USA).

## Results

**Fig. 4:**
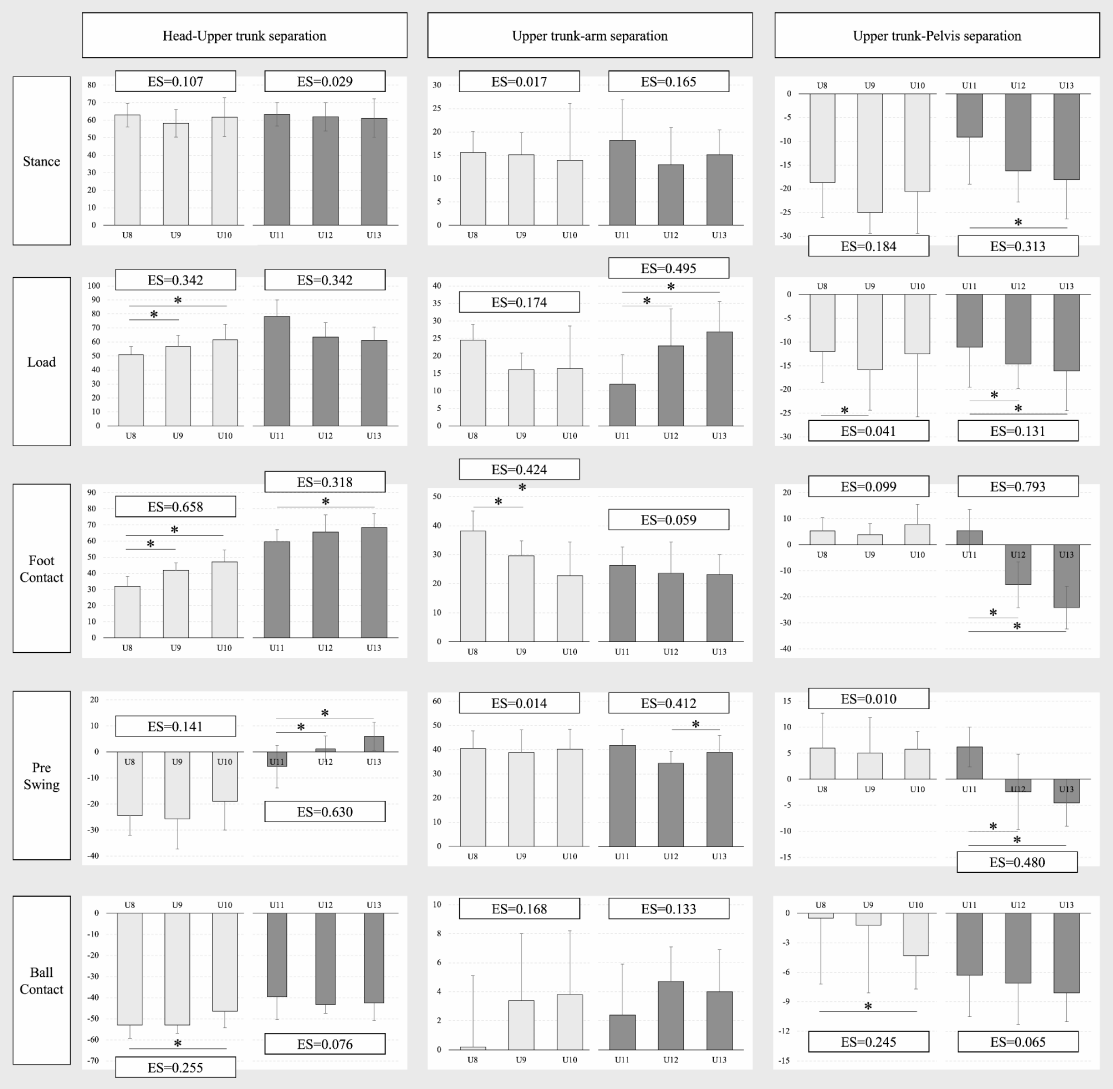
Differences in separational angles during stance, load, foot contact, pre-swing, and ball contact

Chronological age, height, body weight, and competition years were significantly higher for the older age categories in both groups 1 and 2 (Table 1). Table 2 showed the head, upper trunk, and pelvis rotation angles and arm directions for each phase, and Fig. 4 indicated the head-to-upper trunk, upper trunk-to-pelvis, and upper trunk-to-arm separation angles. Several large effect sizes (≥ 0.14) were found in each of the stance to ball contact. Especially, the upper trunk rotation angle was significantly smaller (i.e., rotated toward the catcher) in the foot contact (ES = 0.871 in group1 and 0.497 in group 2) and significantly increased (i.e., rotated toward the pitcher) in the pre-swing phase (ES = 0.221 in group 1 and 0.712 in group 2) as age increased. Regarding the separation angle, the head-to-upper trunk separation angle in the foot-contact phase increased as age increased (ES = 0.658 in group1 and 0.318 in group 2). Furthermore, U12 and U13 showed significantly larger separate angles than U11 not only in the head-to-upper trunk separation angle during the pre-swing (ES = 0.630), but also in the upper trunk-pelvis separation angle during foot contact and pre-swing (ES = 0.793 and 480).

**Table 1:** Basic characteristics of the participants

Variables	Group 1 (N = 17)			Group 2 (N = 13)		
	U8^a^	U9^b^	U10^c^	U11^a^	U12^b^	U13^c^
Age (years)	7.6 ± 0.2	8.2 ± 0.2^*^	9.3 ± 0.2^*†^	10.5 ± 0.3	11.1 ± 0.3^*^	12.2 ± 0.3^*†^
Height (cm)	125.0 ± 5.3	129.0 ± 5.7^*^	133.4 ± 6.6^*†^	140.9 ± 5.5	143.7 ± 5.7^*^	150.2 ± 6.4^*†^
Body weight (kg)	25.8 ± 3.2	28.4 ± 3.5^*^	32.1 ± 5.3^*†^	38.3 ± 6.0	39.4 ± 5.8^*^	44.9 ± 6.2^*†^
Competition years (years)	2.0 ± 0.9	2.2 ± 0.9^*^	3.5 ± 0.9^*†^	4.0 ± 1.9	4.6 ± 2.0^*^	5.7 ± 2.0^*†^

Data are presented as the average ± standard deviation. ^*^Compared with a. ^†^Compared with b (p < 0.05)

**Table 2:** Differences in rotational angles during stance, load, foot contact, pre-swing, and ball contact

Variables	Group 1 (N = 17)				Group 2 (N = 13)			
	U8^a^	U9^b^	U10^c^	ES	U11^a^	U12 ^b^	U13 ^c^	ES
Stance								
Head rotation	26.7 ± 4.7	25.7 ± 3.4	23.1 ± 5.4	0.189	26.5 ± 5.6	26.9 ± 5.4	26.7 ± 3.3	0.004
Upper trunk rotation	-36.2 ± 5.2	-32.7 ± 6.2^*^	-38.7 ± 9.4^†^	0.205	-36.8 ± 5.5	-34.9 ± 9.2	-34.6 ± 7.8	0.041
Pelvis rotation	-17.5 ± 7.4	-7.6 ± 4.9^*^	-18.1 ± 11.6^†^	0.417	-27.7 ± 11.1	-18.8 ± 9.8^*^	-16.5 ± 12.8^*^	0.537
Arm direction	-51.8 ± 4.6	-47.7 ± 5.6^*^	-52.6 ± 14.0	0.099	-55.0 ± 7.2	-48.0 ± 8.4^*^	-49.6 ± 7.6	0.498
Load								
Head rotation	24.3 ± 3.7	25.8 ± 3.4	20.3 ± 10.4	0.191	28.5 ± 6.4	26.8 ± 5.8	27.3 ± 3.0	0.041
Upper trunk rotation	-26.6 ± 4.1	-30.9 ± 6.2^*^	-41.1 ± 13.9^*†^	0.790	-49.7 ± 10.4	-36.8 ± 9.9^*^	-33.9 ± 8.2^*^	0.563
Pelvis rotation	-14.6 ± 6.1	-15.1 ± 6.3	-28.6 ± 17.7^*†^	0.381	-38.6 ± 8.6	-22.3 ± 10.4^*^	-17.8 ± 13.1^*†^	0.714
Arm direction	-51.0 ± 4.6	-51.0 ± 5.6^*^	-57.5 ± 21.4	0.163	-61.6 ± 6.3	-59.7 ± 3.0	-60.8 ± 4.7	0.043
Foot contact								
Head rotation	26.9 ± 4.8	25.9 ± 3.4	21.5 ± 8.1	0.294	29.7 ± 4.7	27.3 ± 5.2	27.8 ± 4.4	0.074
Upper trunk rotation	-5.1 ± 3.5	-16.0 ± 3.9^*^	-25.6 ± 6.8^*†^	0.871	-29.9 ± 5.1	-38.3 ± 10.4^*^	-40.6 ± 6.5^*^	0.497
Pelvis rotation	-10.4 ± 5.7	-19.8 ± 3.3^*^	-33.4 ± 6.5^*†^	0.896	-35.3 ± 8.3	-23.0 ± 9.4^*^	-16.5 ± 8.2^*^	0.699
Arm direction	-43.2 ± 5.0	-45.6 ± 4.8	-48.3 ± 8.0	0.139	-56.3 ± 4.7	-61.9 ± 3.3^*^	-63.8 ± 5.7^*^	0.526
Pre-swing								
Head rotation	27.1 ± 4.8	26.0 ± 3.5	26.1 ± 6.1	0.016	30.3 ± 5.9	30.6 ± 3.7	32.3 ± 4.4	0.068
Upper trunk rotation	51.4 ± 6.5	51.6 ± 10.2	45.0 ± 7.4^*^	0.221	35.9 ± 6.6	29.3 ± 4.7^*^	26.2 ± 3.3^*†^	0.712
Pelvis rotation	45.4 ± 5.9	46.5 ± 7.9	39.3 ± 5.0^*†^	0.381	29.7 ± 5.5	31.7 ± 10.1	30.8 ± 4.7	0.023
Arm direction	11.0 ± 3.4	12.7 ± 4.4	4.9 ± 4.4^*†^	0.498	-6.0 ± 3.3	-5.1 ± 1.8	-12.6 ± 6.2^*†^	0.529
Ball contact								
Head rotation	27.9 ± 4.0	26.1 ± 3.5	29.0 ± 7.2	0.070	33.8 ± 7.9	31.0 ± 3.4	30.8 ± 7.5	0.088
Upper trunk rotation	80.9 ± 3.3	79.2 ± 2.4	75.5 ± 3.1^*†^	0.478	73.5 ± 5.2	74.3 ± 2.2	73.4 ± 2.4	0.022
Pelvis rotation	81.4 ± 3.2	80.4 ± 2.9	79.8 ± 3.4	0.078	79.8 ± 5.5	81.5 ± 4.1	81.5 ± 2.3	0.061
Arm direction	80.7 ± 3.7	75.7 ± 4.0^*^	71.7 ± 4.3^*^	0.539	71.1 ± 3.8	69.6 ± 2.5	69.4 ± 2.5	0.107

Data are presented at the average ± standard deviation of rotational angles (degrees). Effect size (ES) is indicated by the partial η^2^. ^*^Compared with a. ^†^Compared with b (p < 0.05).

Swing velocity increased as age increased (ES = 0.706 in group 1 and 0.686 in group 2); however, the head movement from stance to foot contact (ES = 0.0.188 in group 1 and 0.0.370 in group 2) and that from foot contact to ball contact (ES = 0.436 in group 1 and 0.731 in group 2) decreased with age (Table 3).

**Table 3:** Swing velocity, head movement, and step width during stance, load, foot contact, pre-swing, and ball contact

Variables	Group 1 (N = 17)				Group 2 (N = 13)			
	U8^a^	U9^b^	U10^c^	ES	U11^a^	U12 ^b^	U13 ^c^	ES
Swing velocity (mph)	41.0 ± 4.5	43.6 ± 4.9^†^	46.1 ± 4.8^*†^	0.706	48.9 ± 5.6	53.7 ± 6.9^*^	59.0 ± 8.8^*†^	0.686
Head movement ST to FC (cm)	11.2 ± 2.4	11.3 ± 2.7	9.3 ± 2.4^*†^	0.188	9.9 ± 1.7	7.8 ± 1.9^*^	8.2 ± 1.4^*^	0.370
Head movement FC to BC (cm)	7.8 ± 1.0	8.5 ± 1.2	6.5 ± 1.1^*†^	0.436	6.2 ± 1.2	4.7 ± 1.0^*^	3.8 ± 0.6^*†^	0.731
Step width at Stance (cm)	40.5 ± 3.0	38.7 ± 2.8	41.8 ± 2.8^†^	0.300	44.1 ± 3.3	45.8 ± 3.0	47.0 ± 3.7	0.166
Step width at FC (cm)	49.1 ± 5.3	50.4 ± 3.9	52.8 ± 4.3^*^	0.219	56.0 ± 5.1	59.0 ± 4.6	59.7 ± 4.1	0.180

Data are presented as the average ± standard deviation. Effect size (ES) is indicated by the partial η^2^. ^*^Compared with a. ^†^Compared with b (p < 0.05). BC, ball contact; FC, foot contact; ST, swing timing.

## Discussion

This study was performed with a focus on longitudinally determining when and how batting kinematics changes occur within individuals during youth. We hypothesized that the ability to separate the pelvis, upper trunk, and arm segments temporally and spatially during the batting motion would improve as age increased. However, the relationships between age and body size did not differ significantly from that observed in previous studies of Japanese youth [17, 18].

The transitional movement refers to the stance-to-foot contact, during which the weight transfer to the stepping leg occurs after the weight is briefly on one leg during the pivot. Furthermore, the load is important for smooth transitional movement because of energy accumulated in the lower extremities and trunk and is necessary to obtain high bat head velocity on ball impact by using a kinetic link [3]. During the study period, the upper trunk and pelvis rotation angles gradually increased toward the catcher from the U8 to U11; however, U12 and U13 showed less rotation toward the catcher. Dowling and Fleisig [6] found that the pelvic rotation angle of youth players (age, 11.8 ± 1.1 years) during load was rotated more toward the catcher than that of professional adult batters (age, 20.2 ± 2.3 years). Tsutsui et al. [19] investigated the relationship between the lower extremity muscle function and swing velocity, which often indicates the batting performance, and reported that the hip internal rotation torque in the pivot leg was related to swing velocity for players < 9.5 years. However, the modified star excursion balance test of the pivot leg showed that the overall lower extremity function was related to that of players > 9.5 years. This is possible that because younger baseball players do not have sufficient lower extremity function in the pivot leg; hence, they try to improve swing efficiency by compensating and preparing for energy accumulation during the translational movement phase with trunk rotation toward the catcher.

Posture or movement during preparation and subsequent body weight transfer should be emphasized to enable a powerful bat swing [20]. Foot contact corresponds to the point at which the motor energy accumulated in the pivot leg during load switches to the stepping leg. Furthermore, [21] reported that shifting the body weight to the stepping leg too early would cause the ground reaction force to counteract the lower body movements, resulting in a less powerful bat swing. Additionally, they considered well-coordinated weight transfer from the pivot leg to the stepping leg was regarded as the key to powerful swinging. In our study, as age increased, the upper trunk and arm direction angles increased toward the catcher, and the head-to-upper trunk separation angle widened during foot contact despite weight transfer toward the pitcher. Furthermore, the head movement distance from stance to foot contact was larger in the U8 and U9 and smaller in the U12 and U13. Notably, batters need to keep their head in space to minimize eye movement and accurately impact the pitched ball [22] while shifting their weight toward the pitcher from load to foot contact. Moreover, the upper trunk, pelvis, and the arm with the bat need to be twisted toward the catcher once to make a strong swing, which is likely to blur the vision. Therefore, with age, the player may acquire a batting strategy in which the trunk is sharply rotated while the head is kept in space. Interestingly, from load to foot contact, the upper trunk rotation angle changed toward the pitcher from the U8 to U11; however, of the U12 and U13 changed toward the catcher. Additionally, the head-to-upper trunk and upper trunk-to-pelvis separation angles began to decrease during the transitional movement phase, suggesting that younger batters, until approximately 10–11 years perform early trunk rotation as they chase the thrown ball.

The upper trunk rotated toward the pitcher relative to the pelvis up to the U11 and toward the catcher relative to the pelvis in the U12 and U13. This tendency affected the ball contact, with the U8 exhibiting a narrower upper trunk-to-pelvis separation angle than the U10. Notably, the lower extremity and trunk provide a foundation for the kinetic chain [10, 23] and contribute approximately 54% of the total force developed during a tennis serve—a rotational motion similar to baseball batting. In addition, when the trunk movement exerts its maximum power, it is predicted that the ball will be hit more strongly if the extra power to twist the trunk is still reserved during ball contact. Moreover, the average trunk separation angles of collegiate and professional players shown by previous studies were − 10° and − 13°, respectively, with suppressed torso rotation. Younger batters had greater pelvis velocity than adults [7], high school players, and professional batters [6]. This may be because young batters have a smaller moment of inertia in their body, enabling them to separate and move segments faster. Therefore, the U8 and U9 may use the pelvis and trunk muscle strength during rotation to compensate for the faster pelvis rotation. Therefore, careful observation of the young batter’s characteristics is necessary to avoid excessive upper torso rotation against the pelvis after foot contact in the batting motion.

The strength of this study was its longitudinal design. Individuals were examined for three seasons. Based on this study`s findings, coaches and managers could determine the developmental stage of baseball batting motion by observing the angle of rotation of the head, upper trunk, pelvis, and arm direction and the separation angle of each relative to the home plate. However, the study had some limitations. First, the difference between group 1 and 2 attributes cannot be completely ruled out, especially because we conducted measurements during only three seasons due to the coronavirus disease 2019. Therefore, the change between the U10 and U11 should be interpreted cautiously. Second, we used a toss machine to standardize certain aspects of the swing; however, its application to batting may be different because it ignores that batting during a game is performed relative to a pitcher’s throw. Nevertheless, we believe that we can evaluate the reaction to the projected ball and the function of coordination more comprehensively than the conventional method of measuring hitting using a tee stand. This will enable more generalizations about the developmental process of the batting motion of young baseball players. Lastly, unlike a laboratory study, we could not determine many joint coordinates because many target batting motions were measured on the field every season. Although previous studies have shown that youths have less back elbow flexion and greater back shoulder abduction than adults, we could not clarify the kinematics of the upper and lower extremities. Therefore, it is necessary to investigate the kinematic parameters of the whole body to clarify the developmental process of more detailed batting motions.

## Conclusions

We longitudinally identified age-related changes in batting kinematics, such as rotational and separational movements of the head, upper trunk, pelvis, and arm direction in youth baseball players. We observed many changes in batting kinematics among each age group. Notably, the upper trunk-to-pelvis separation angle at foot contact and pre-swing showed larger in U12 and U13 than U10; therefore, the thoracolumbar area, while swinging, can be separated after approximately 10–11 years of age. In addition, older batters had a larger separational movement between the head and upper trunk during foot contact and the pre-swing, while U8 batters had a smaller separation between the upper trunk and pelvis during ball contact. Based on the above findings, it would be important for the instruction of younger baseball players to understand the underdevelopment of trunk separation when batting and encourage the acquisition of such separation movements.

## Data Availability

The datasets used and/or analysed during the current study are available from the corresponding author and the director of baseball league on reasonable request.
